# A mixed-methods study of the effectiveness and perceptions of a course design institute for health science educators

**DOI:** 10.1186/s12909-022-03910-w

**Published:** 2022-12-16

**Authors:** Julie Speer, Quincy Conley, Derek Thurber, Brittany Williams, Mitzi Wasden, Brenda Jackson

**Affiliations:** 1 A.T. Still University, Mesa, AZ USA; 2 Arizona State University, Tempe, AZ USA

**Keywords:** Course design, Faculty development, Health sciences, Higher education, Mixed methods

## Abstract

**Background:**

Most health care professionals get their start in academics without formal teaching training. As such, institutions encourage participation in opportunities to address gaps in faculty’s knowledge of pedagogy and learning theory in order to promote both successful student and patient outcomes. This study aimed to examine the reception of a faculty development program focused on teaching participants the basics of course design.

**Methods:**

Applying a mixed-method approach, this retrospective study used pre/post-tests, assignment grades, self-assessment questionnaires, and focus groups to elucidate the impact of the faculty development intervention on course design. The participants (*n* = 12) were health educators from a private all-graduate level university with campus locations across the United States, including in the Southwest and Midwest. In the Course Design Institute (CDI), the participating faculty learned evidence-based instructional approaches and techniques to implement contemporary teaching practices.

**Results:**

The data from the pre/post-tests and focus groups suggest that participants learned about topics including instructional alignment, learning goals and objectives, instructional strategies, assessment planning, feedback approaches, communicating expectations, and adult learning theories by participating in this course. The final deliverable scores indicate that the CDI graduates were able to apply a backward design process to plan their own instruction. Data from both the survey and the focus groups suggest that participants were satisfied with the experience and particularly appreciated that the course was relevant to them as educators in the health sciences.

**Conclusions:**

The results of this study indicate that the CDI was influential in developing the faculty’s knowledge of the course design process, promoted the application of course design and pedagogy skills amongst CDI graduates, and positively impacted self-reported attitudes about their teaching abilities. In addition, feedback from participants indicates that they recognized the value of this program in their own development and they believed it should be a required course for all educators at the institution.

**Supplementary Information:**

The online version contains supplementary material available at 10.1186/s12909-022-03910-w.

## Background

### Issues in Healthcare Education

Healthcare practitioners who choose to take on faculty roles in higher education are valuable mentors due in part to their strong clinical skills and diverse workplace experiences. The assumption is that these new educators will be immediately prepared to transfer their expertise to the next generation of professionals. However, it is generally known that there is a difference in the skills required to be an expert and to teach others to become experts. In fact, many clinicians begin a career in academics without formal training on how to teach; often finding themselves unprepared for the challenges of their new roles as educators [1–4].

A lack of teaching skills has significant consequences for students and faculty members. Students may be adversely affected in their learning experiences, especially in the development of critical thinking and problem-solving skills, which current educational standards require to be demonstrated within the educational process [5–8]. Furthermore, in order to help students efficiently and effectively develop essential clinical and other desirable skills, faculty must understand the cognitive process and utilize current instructional methods. The lack of pedagogical training for new faculty may also affect their job satisfaction and retention; leading to frustration and burnout [9–11]. Health science faculty may face significant challenges when learning how to teach on the job and through trial and error [9, 12]. Without support from faculty development programs, educators may struggle to adopt contemporary pedagogical methods in their classrooms [13]. More specifically, they often fail to establish comprehensive learning goals that are tightly aligned with clear and measurable learning objectives [14]. They may also struggle to understand the implications of critical situational factors (elements and factors of the learning situation such as the number of students, the time and duration of the class, the class subject, characteristics of the learners and instructor(s), and expectations of external groups including accreditation organizations) and their influence on the learning context of their courses which can inadvertently create barriers in their student’s learning experience [15]. Faculty without foundational knowledge in teaching and learning often misalign assessments with the learning objectives, widening the gap for students to apply what they intend to learn in authentic settings [11]. Without connecting the assessments with learning objectives, it is unlikely that instructors will be able to determine what knowledge and skills students have gained from completing the course. Similarly, faculty may overlook the issues caused by not developing the appropriate learning activities to support the achievement of the stated learning objectives [7, 10]. As a result, students are often left on their own to learn the content. Or, even more detrimental to students’ learning, the instructional activities might not adequately prepare them for high-stakes assessments [16]. In sum, the basic formula for effective instruction – the intentional alignment between the learning goal and objectives, learning activities, and assessments (both formative and summative) – is often missed.

### Faculty Development in Healthcare Education

To address gaps in health sciences faculty’s formal training in pedagogy and learning theory and to promote both successful student and patient outcomes, departments, institutions, and intramural organizations have created faculty development programs [5, 17–20]. These exist across a broad spectrum ranging from mandatory to voluntary, short-term (a single event or short series of events) to long-term (longer than a year), and may be either discipline-specific or interdisciplinary [11, 18, 19, 21–23]. While the broader goals of these programs remain relatively consistent, the specific objectives may vary. Common themes include improving teaching effectiveness and promoting (both general and specific) learner-centered instructional approaches [5, 17, 18, 22], designing courses and developing curricula [17], providing feedback to students [17], and establishing faculty learning communities (FLCs) [5, 23]. McLean et al. described over a decade ago that there had been a progressive shift within the health sciences towards the rigorous evaluation of faculty development programs [19]. Common outcomes have included increased faculty confidence, use of student-centered approaches, empathetic instruction, and reflective teaching [18, 22, 24]. Although it is difficult to determine the precise impact of faculty development programs on the long-term development of educators’ skills, student learning and retention, and ultimately patient outcomes, the effects of faculty development have nonetheless been described through both qualitative and quantitative data and may be most profound for educators early on in their transition from clinic to the classroom [11, 19, 21, 22, 24].

### Training Faculty on the Course Design Process

One faculty development opportunity that has been implemented across a variety of institutions is an intervention often referred to as a Course Design Institute (CDI). A CDI typically provides a practical learning experience to introduce faculty to the principles of backward design. The motivation is that establishing a foundation in course design principles can empower educators to design effective courses that enable them to achieve the ultimate goal of delivering significant and impactful learning experiences for students [15, 25].

While the format of the CDI is often customized to the particular university or program, it is typically designed to offer an iterative, structured experience whereby participants learn about course design processes and principles. Over multiple days or weeks, a cohort of educators follows established methods to craft measurable learning objectives, design assessments and content, and select instructional feedback approaches to help students achieve the targeted course goals [13, 14, 26]. The CDI also provides opportunities for the participating educators to brainstorm and discuss best practices together and to receive feedback from their colleagues and the leaders of the CDI (who may be faculty, instructional designers, or other teaching and learning experts).

### Educational Theoretical Framework

Backwards design is an effective and widely used instructional design method that is often taught in CDIs [25]. Put simply, backwards design follows a three-stage process by which courses are designed by starting with intended outcomes and working backward from there. The stages, in order, are (1) identifying desired results, (2) determining acceptable evidence, and (3) planning learning experiences and instruction [25]. Though this concept is not new, Wiggins and McTighe formalized this model in their seminal work *Understanding by Design* [25]. They emphasized developing an understanding of “big ideas,” which they defined as “a concept, theme, or issue that gives meaning and connection to discrete facts and skills” (p. 5) [25]. They held that when instruction was focused on big ideas, it would center on the learner (i.e., what do students know?) rather than the instructor (i.e., what do I teach?) [25].

For this reason, backward design has been widely used and adopted in many contexts including health sciences education [6, 15, 27, 28]. For example, Emory described how a backward design approach was employed at the University of Arkansas to transform a nursing course that taught students how to apply medical concepts to professional practice [27]. Just as the backwards design process has been used to develop individual courses, it has also been implemented when planning instruction at the program level. For example, Wright et al. found that the backward design approach was helpful when redesigning the pharmacy education program at Auburn University [28].

### Overview of the Present Study

The purpose of this study was to evaluate the efficacy and impact of a Course Design Institute (CDI) training program implemented at a medium-sized health science university in the United States. The CDI, which was offered three times between May 2020 and August 2021, was customized to meet faculty development needs and to provide guidance on effective course design. Specifically, this study was designed to elucidate (1) what new knowledge faculty acquired about course design, (2) what skills related to course design and pedagogy were developed, and (3) faculty’s attitudes related to their teaching practices and their experiences participating in the CDI program.

## Methods

Applying a mixed-method approach, this retrospective study used performance scores, perception data, and focus groups to assess the impact of a faculty development intervention focused on course design. In addition, quantitative data from learning tests, assignment grades, and self-assessment questionnaires were analyzed in order to determine participants’ course design knowledge, skills, and attitudes. Finally, qualitative data were also analyzed for themes related to the reception and outcomes of the faculty development program.

### Overview of the Course Design Institute (CDI)

The CDI model described herein was adapted from existing programs [13, 14, 26, 29] and sought to provide a professional development opportunity to an interdisciplinary cohort of health educators employed by a small all-graduate level health sciences university. The course spanned seven weeks and participants engaged in synchronous online meetings via Zoom video conferencing (Zoom Video Communications; San Jose, California) for 1.5 hours per week. All participants applied to be accepted into this voluntary course regardless of their prior teaching experience, course length, and instructional teaching modality (in-person, online, hybrid, clinical). Instructional designers and faculty development experts taught the CDI and took a systematic and facilitated approach to introduce the participants to the subject of learner-centered, backward course design. Weekly topics included creating measurable learning goals and objectives, selecting appropriate instructional strategies, creating a plan for instructional content, aligning assessments, and giving and receiving feedback. Participants were assigned weekly homework activities such as quizzes, discussions, and writing assignments to reinforce the course design concepts. For the culminating activity, each participant completed a learning artifact called the Course Design Blueprint to demonstrate their course design skills (Additional file 1). Participants iteratively developed their blueprint which served as a comprehensive proposal to communicate the educator’s plan for their course and the methods by which they sought to promote student learning outcomes. As they developed their course design plan, each member of the CDI cohort received personalized feedback from their peers and the course instructors. Additional long-term goals of the CDI were for participants to be able to repeat the process, utilize the backwards design framework when designing other instruction, evaluate the components of their teaching and learning practices, and make evidence-based pedagogical decisions to best support their learners.

### Participant Recruitment and Research Study Process

To explore the impacts of the CDI program on participants’ knowledge, skills, and attitudes, graduates of the program across the three cohorts from Summer 2020 to Summer 2021 were invited via e-mail to enroll in the IRB-approved study (IRB #2022–060). The only requirement for inclusion was the completion of the CDI. CDI participants who started the course but did not successfully finish it were excluded because the amount of the course they experienced varied significantly. The recruitment materials explained the purpose of the study, risks and benefits, and compensation. Those who enrolled were provided links to participate in a post-course research survey (Qualtrics; Provo, UT) and an online synchronous focus group. Study participants were also informed that educational artifacts they had previously submitted during the CDI would be evaluated for this study. Twelve CDI graduates enrolled and answered the research survey, and from this participant pool, ten chose to attend a focus group session. The demographic information of the participants is presented in Table 1.

**Table 1:** Summary of demographic information of participants

Demographic Factors	*n* (%)
Age	
Average	41.9
Range	32–53
Gender Identity	
Female	10 (83.3)
Male	0 (0)
Other or Preferred Not to Answer	2 (16.7)
Highest Education Level	
Doctorate	7 (58.3)
Masters	5 (41.7)
Bachelors	0 (0)
High School	0 (0)
Employment Status	
Full-time	11 (91.7)
Part-time	1 (8.3)
Years Teaching Experience	
Average	6
Range	0–20
Experience by Teaching Format (including before and after onset of COVID-19)	
Face-to-Face	10 (83.3)
Online	10 (83.3)
Hybrid	8 (66.7)
Health Science Profession	
Audiology	1 (8.3)
Dental Medicine	2 (16.7)
Osteopathic Medicine	1 (8.3)
Occupational Therapy	3 (25)
Physical Therapy	3 (25)
Public Health	1 (8.3)
Other	1 (8.3)

### Materials and Instruments

#### Pre−/Post-Tests Examining Pedagogical Knowledge Gains

To examine the impact of the CDI on the participating educators, a pre-post style assessment was administered which measured knowledge gained as a result of participating in the course. The tests were delivered immediately before and after the course and aimed to evaluate participants’ grasp of foundational course design concepts. The knowledge-based questions were in the form of multiple-choice, fill-in-the-blank, and short-answer questions. Additional questions asked about perceptions of self-efficacy, particularly as it related to their ability to design and facilitate a learning-centered, evidence-based course. This approach, including the incorporation of self-reported outcomes, has been extensively used in teaching and learning studies to measure changes in knowledge, attitudes, and behaviors and has also been previously used to evaluate the consequences of faculty development programs [13, 30].

#### Course Design Blueprint Scores

Participants’ final scores on their culminating project (the Course Design Blueprint) were evaluated to measure course design skills developed by the end of the CDI. The course instructors assessed these documents following a predetermined grading rubric (maximum score = 20 points; Additional file 1) which reflected evidence-based principles of sound course design.

#### Teaching Appraisal Inventory

A modified version of the Teaching Appraisal Inventory (TAI) used by Palmer et al. [13] was administered through the post-course research survey to assess the participants’ self-efficacy toward teaching concepts. The TAI was first developed by Balam [31] as a 46-item instrument aimed at uncovering instructors’ confidence in classroom teaching practices. Each item asked participants to rate their perceived confidence in a statement starting with “I think I can …” followed by a common classroom teaching practice on a 7-point Likert-type scale (i.e., 1 = Strongly disagree, 2 = Disagree, 3 = Somewhat disagree, 4 = Neither agree nor disagree, 5 = Somewhat agree, 6 = Agree, 7 = Strongly agree). Palmer et al. modified the instrument to group the 46 practices into seven overarching categories, used as subscales for measurement: goals and objectives, assessment, classroom management, learning activities, class facilitation, effective assignments, and overall teaching [13]. The version of the TAI used in the present study was modified slightly to reflect the content of the present CDI (Additional file 2). Nevertheless, the instrument used the same categories identified by Palmer et al. [13].

#### CDI Satisfaction

The post-course research survey included a combination of two Likert-type, two open-ended questions to assess participants’ satisfaction with the CDI, and demographic questions. The ratings from the Likert-type questions were used to measure how well participants enjoyed the class. Additionally, responses to the open-ended questions were analyzed by two authors (QC and BW) and coded for themes of what participants enjoyed most and least about the class.

#### Focus Groups

Focus groups were utilized to assess participants’ attitudes toward implementing course design principles into their teaching practice [11, 21, 32]. The three focus groups were comprised of 2–5 participants each, grouped by availability rather than discipline or department, and were conducted using the virtual conferencing platform Zoom [11, 33, 34]. All focus groups were led by the same individuals (JS and DT) and followed identical protocols. Before the focus group, participants were instructed to review their course design blueprints from the CDI to prepare for the discussion. Upon entry into the Zoom room, participants were briefed on the purpose of the focus group and guidelines for the discussion. They were also informed that the session would be recorded for research purposes. Authors (JS and DT) then facilitated a 50–60-minute discussion, starting with general questions that became more specific throughout the session; follow-up questions were asked when needed to prompt participants to expand on their thoughts or experiences [34, 35]. The focus group questions (Additional file 2) were designed to promote discussions about faculty’s teaching experiences since the CDI, how the CDI has informed their teaching practice, and the opportunities and barriers faced when making changes to the design of their courses.

Transcripts of the recorded focus groups were auto-generated via Zoom and inspected and edited for accuracy. The data were then analyzed using a descriptive coding technique using Atlas.ti software (ATLAS.ti Scientific Software Development GmbH; Berlin, Germany) to identify emergent themes and assign responses to one or more respective categories [11, 36]. Three research team members (QC, JS, DT) independently conducted a first pass of the coding and then examined their results for agreement. A codebook was then developed and used to complete the qualitative data. Anonymized quotes were also identified that corresponded to particular themes and provided more context for the responses [13, 32, 37].

#### Data Visualization and Statistical Analysis

Unless otherwise noted, the data were analyzed and visualized using GraphPad Prism (version 9.4.1; San Diego, CA). One-way unpaired t-tests were used to evaluate the hypothesis that participation in the CDI would contribute to an increase in knowledge related to teaching and learning topics. Descriptive statistics including the average blueprint score and range amongst the participants were calculated to examine the ability of CDI graduates to apply the backwards design process to their own instruction. Cronbach’s alpha analysis was conducted in SPSS to determine the internal consistency of the responses to the modified TAI instrument and mean and standard deviations of scores in the sub-scales were calculated to analyze the self-efficacy data. Furthermore, the mean and standard deviations to the Likert-type survey questions were calculated to quantify these numerical results. Lastly, intraclass correlations (ICC) were calculated in Excel software (Microsoft, Redmond, WA) to determine the interrater reliability of the categorization of the open-ended survey question and focus group responses [38]. The ICC technique was used as it is an approach for two or more raters without absolute agreement [39].

## Results

### Pre- and Post-tests

**Fig. 1:**
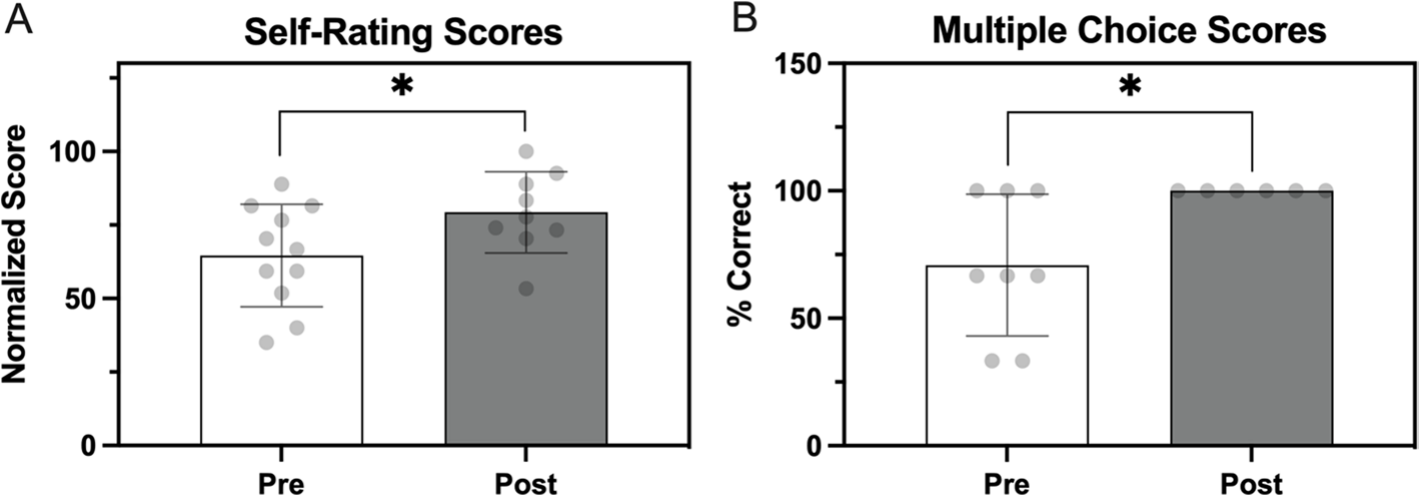
Results of pre- and post-tests which contained Likert-type questions about perceptions of participants’ course design knowledge (A) and multiple-choice questions on pedagogical principles (B). For both plots, bars = mean ***±*** standard deviation, each dot = data from 1 participant, * *p* < 0.05; unpaired *t*-test

The results from the course knowledge assessment, as measured by a pre- and post-test (Fig. 1), indicated statistically significant learning gains due to participation in the CDI. Immediately following the course, respondents showed increased ratings of their own knowledge of course design principles (Fig. 1 a, *p*= 0.03) as well as an increased number of correct responses to multiple choice questions related to pedagogy (i.e., “What is the difference between formative assessment and summative assessment?”**;** Fig. 1 b, *p* = 0.01).

### Course Design Skills

**Fig. 2:**
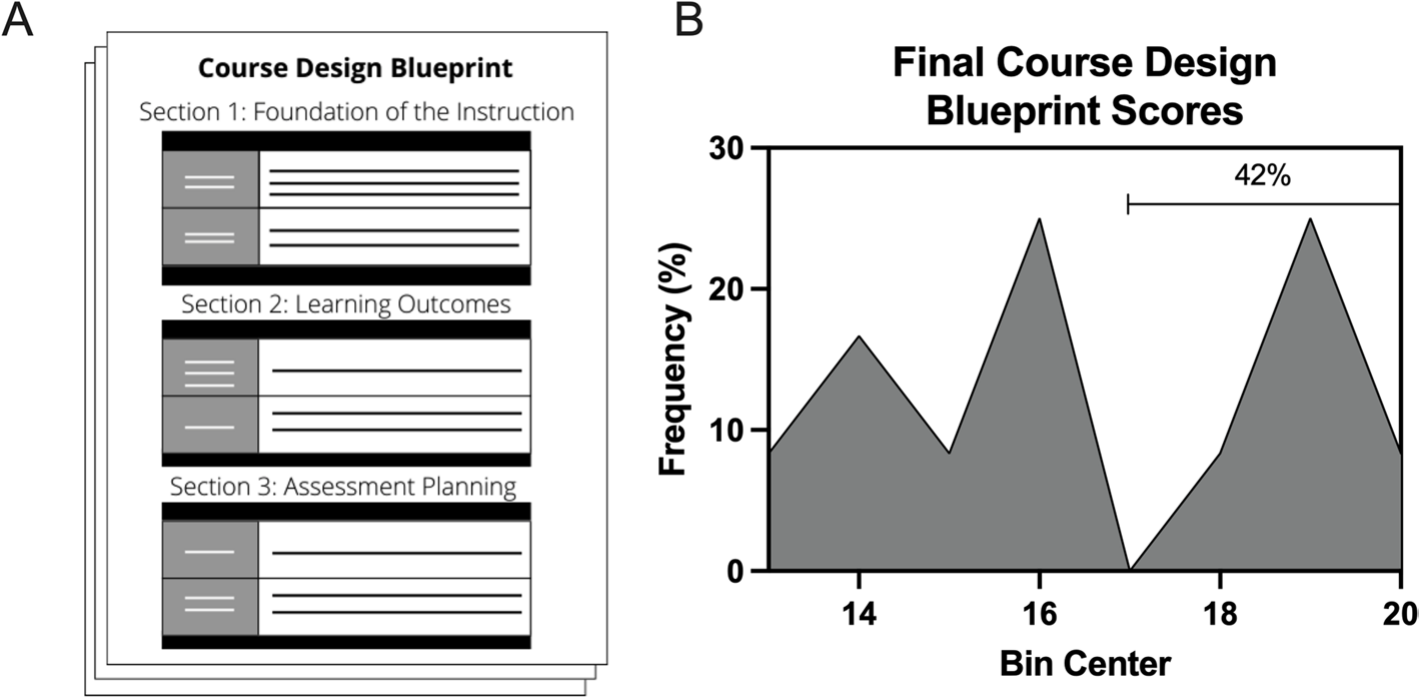
The Course Design Blueprint contained sections that helped scaffold the course design process and contained prompting questions to which the participants responded (A). The final blueprints were graded using a rubric with a maximum score of 20 (*n* = 12*;* B)

Amongst CDI participants who completed the program, as measured by the course design blueprint (Fig. 2 a, Additional file 1), the course design skills scores ranged from 13 to 19.50. The average score was 16.57 (SD = 2.30) with 42% of the scores falling between 17 and 19.50 (Fig. 2 b).

### Teaching Appraisal Inventory as an Assessment of Self-efficacy

**Fig. 3:**
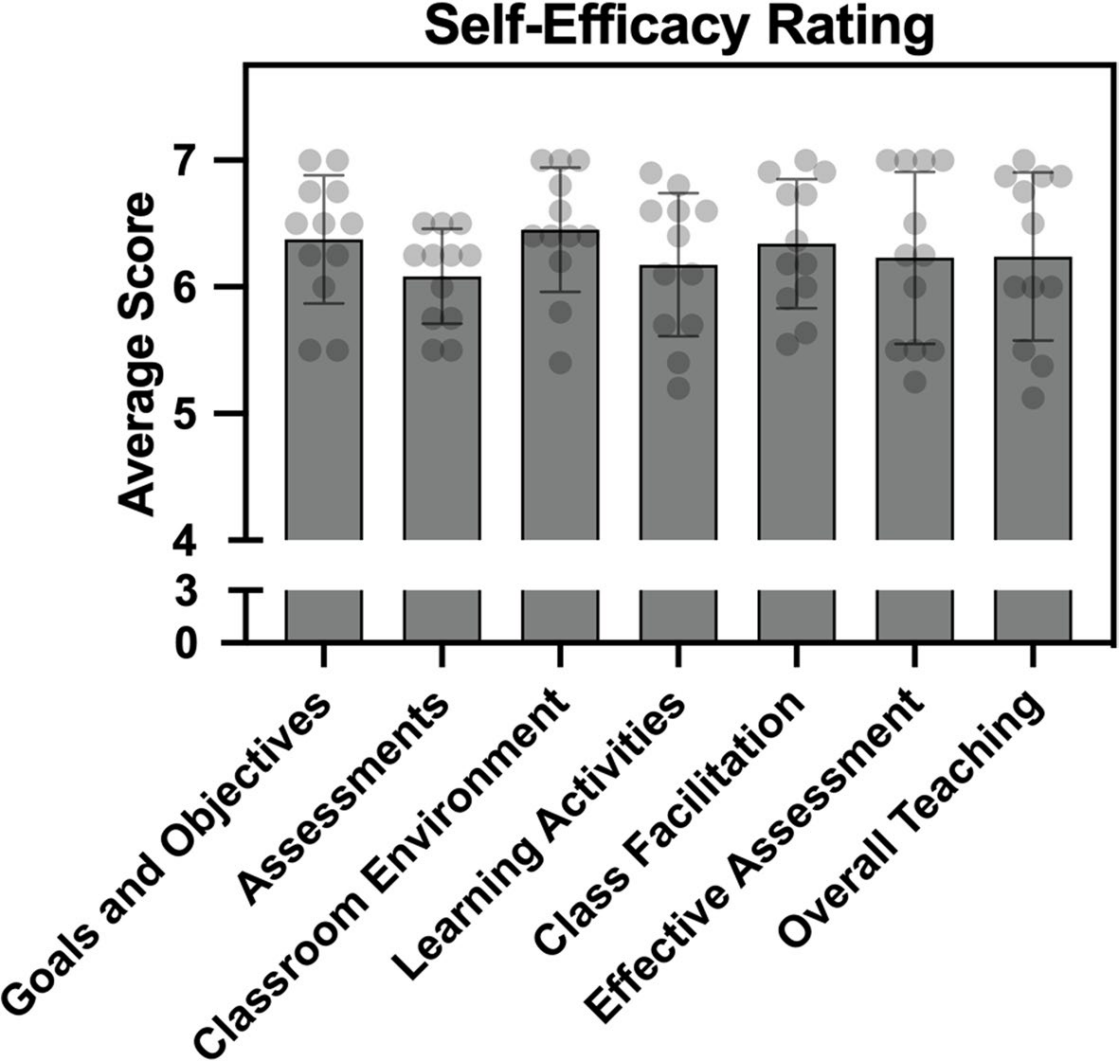
A Teaching Appraisal Inventory (TAI) was used to measure self-efficacy across 7 sub-scales. Bars = mean ± standard deviation, each dot = data from 1 CDI participant

On a scale from 1 (strongly disagree) - 7 (strongly agree), participants’ perceived self-efficacy in their classroom teaching practices averaged 6.27 (*SD* = 0.26; Fig. 3 and Additional file 3). The classroom environment subscale had the highest average score of 6.45 (*SD* = 0.44), while the assessment subscale had the lowest average score of 6.08 (*SD* = 0.20). A high overall Cronbach’s alpha (α = 0.96) determined the strong reliability of this instrument in the context of this study.

### Course Satisfaction

**Fig. 4:**
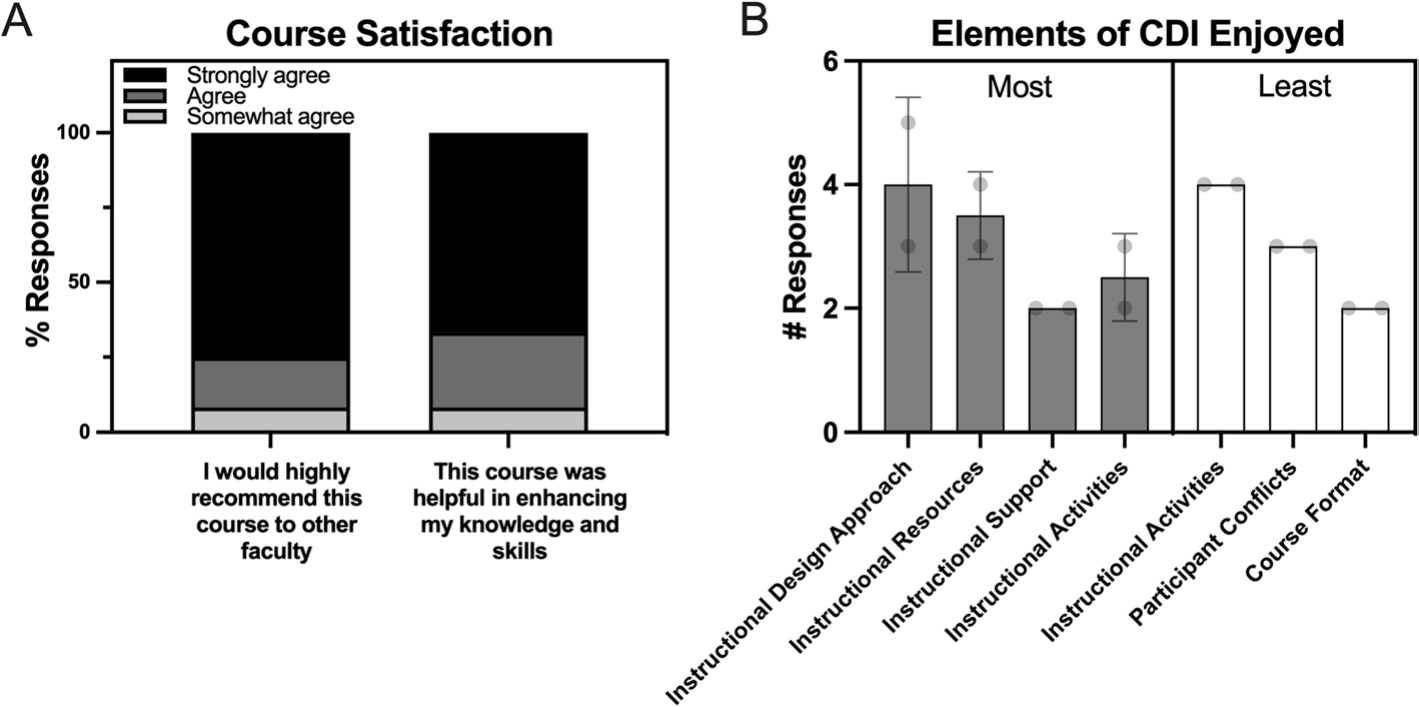
Responses (*n* = 12) to Likert-style questions on course satisfaction (response options ranged from strongly disagree to strongly agree (A). Responses (*n* = 12) to open-ended questions on the most and least enjoyed elements of the CDI were coded into categories (B; bar = mean ± standard deviation, each dot = number of quotes assigned by each coder)

One hundred percent of participants indicated that they would recommend the course to other faculty and that they found the course to be helpful in learning new knowledge and skills (Fig. 4a). On a scale of 1 (strongly disagree) to 7 (strongly agree), the average responses to these statements, “I would highly recommend this course to other faculty” and “This course was helpful in enhancing my knowledge and skills in developing an evidence-based practice of course design” were 6.67 and 6.58 respectively.

Data from open response questions were coded by two authors (*ICC = 0.54*) and indicated the aspects of the course that participants liked most and least (Fig. 4 a, Table 2). In particular, participants indicated they enjoyed the instructional approach used to facilitate the CDI and found many in-class activities, resources, and instructional support to benefit them. In contrast, participants shared how they least enjoyed other instructional activities such as discussion boards, how they encountered scheduling conflicts or external barriers to their learning process, and preferences related to the course format, particularly the duration and mode of the class.

**Table 2:** Responses to open-ended questions, “What did you enjoy the most about the CDI?” and “What did you enjoy the least about the CDI?”

	Category	Example Quote(s)
Enjoyed Most	Instructional Design Approach (practical, relevant, personalized, etc.)	“The information that I was learning was able to be implemented in my class right away”
	Instructional Resources (blueprint, educational technology, etc.)	“The Blueprint as a guide to course design” “The learning management system aspect as I became more competent in using LMS (Canvas)”
	Instructional Support (instructor responsiveness, timely feedback, office hours, etc.)	“I loved how enthusiastic and engaging the TLC instructors were while teaching the course …” “Feedback from TLC and peers, designated times to meet each week...”
	Instructional Activities (homework, in-class activities, practice, etc.)	“The opportunity to try the different strategies. I also liked the collaborative nature of the course”
Enjoyed Least	Instructional Activities (discussion board, too much material covered, etc.)	“… I was a little overwhelmed creating an entire course …” “The discussion posts. I’m just not sure I got as much out of them as I should have. It was great to see other participant’s blueprints.”
	Participant Conflicts (time, motivation, etc.)	“… It seemed hard to make it to all of the meetings, but I liked that there was the built-in accountability …” “Lack of time flexibility required for synchronous course sessions.”
	Course Format (how and when class sessions were held)	“It was too short, and I felt I didn’t have time to master understanding of the different methods and strategies” “If I had to choose, it would be the fact that we met on Zoom. I’m always going to be a face-to-face person, but I understand that it is important to open the opportunity to employees/faculty of all...campuses …”

### Focus Group

**Fig. 5:**
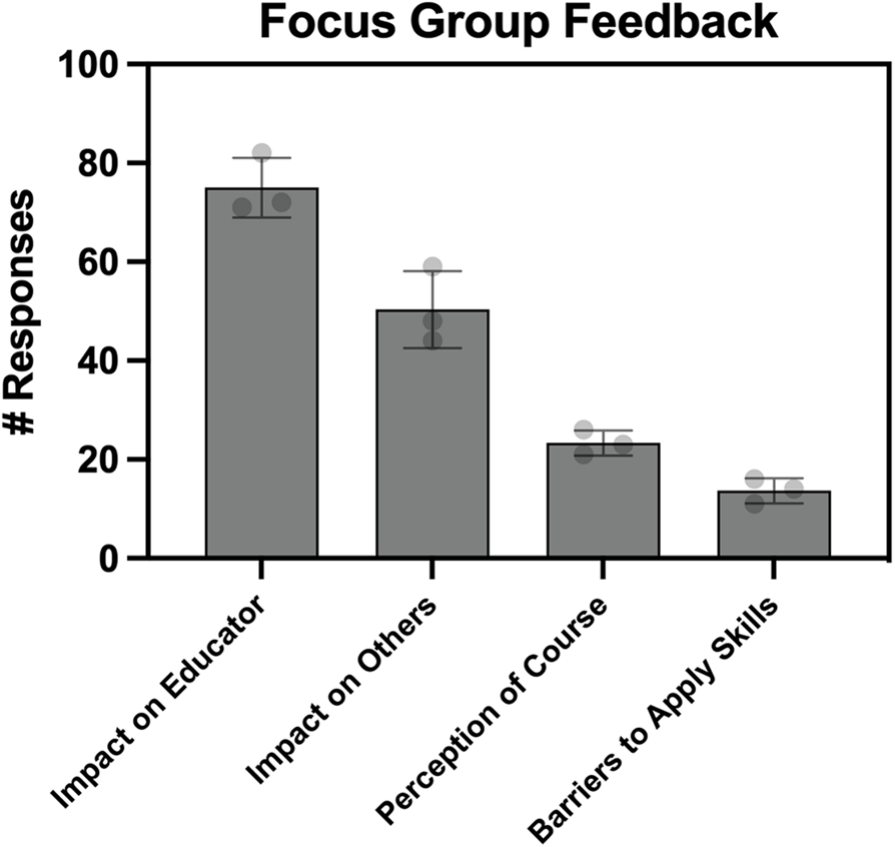
Transcripts from the focus groups were examined for common themes and quotes were coded into thematic categories (bar = mean ± standard deviation, each dot = number of quotes assigned to a given category by each coder)

Qualitative feedback from the focus group participants was coded by three authors (*ICC* = 0.92) and was found to fall into four major categories: 1) the impact of the CDI course on the participants themselves, 2) the impact of the CDI on others, 3) the perception of the CDI, and 4) barriers encountered when applying course design skills learned in the CDI (Fig. 5, Table 3).

**Table 3:** Example quotes by thematic categories from the focus group responses

	Category	Example Quote
Impact on Participating Educators	New knowledge and skills participants developed through the CDI (practical and theory)	“… it really helped to highlight your assessments and lining them up with your objectives …”
	Advocating for student-centered learning (articulating, justifying, and advocating for course design decisions)	“… I’ve never had to defend my instructional strategy before and like I was really pushed in that way …”
	Improving usability (seeing course from student perspective, improving course based on that information)	“… [T] he main thing was having a user-friendly platform for the students, so I benefited very well from the canvas instructions and now, if you look at my pages for the course are very well structured …”
	Personal development (growth, fulfillment, motivation)	“… this course gave me the courage and the validation..”
Impact of the CDI on Other Educators and Courses	Transferability of the course design process (applying what was learned to other courses or sharing the process with other educators)	“… [My goal is] to use this blueprint for other courses that I teach because I find it to be so effective and so helpful in looking at the whole picture …”
	Student experience/outcomes (impact of CDI and new skills on student learning or experiences)	“… I feel like their ability to retain the skills that they learn in my class has improved …” “… some students shared with me that they became better test takers by taking the course …”
	Recommending CDI for other educators	“… we should all be required to take the course …” “… I think it’s like such a great service for the employees here to become better teachers …”
	Interacting with others (opportunities for participants to give feedback to their peers in CDI)	“… I was able to interact with faculty from … different schools … it was neat to see the things that they were learning and teaching and the friendships that were developed … they’re trying to become a better teacher and trying to get a little bit of advice on how they can do a better course …”
Perception of the Course	Comparison to other faculty development opportunities	“… I got my post-professional OTD and we covered some but not to the depth of what CDI did. I got very surface level information on how to possibly teach and academics …” “… I do have a master’s in education, but even then I learned some things about technology, when I was in that. But I still didn’t learn all the details that I learned …”
	Opportunities for improvement of CDI	“… Maybe you can have in the future, like those who graduate you can have a community of practice …”
Barriers to applying course design skills	Human Capital	“… the main thing was just time constraints and having to prioritize other parts of my job …”
	Departmental Resistance	“… I’d say the barriers were more cultural … you know just because that’s not necessarily the practice for the department …”
	Budget	“… something that I had to think about was budget and because budgets have been tough since COVID and since enrollment is down all over the country …”

On average, across the raters, the most common responses were attributed to describing the impact of the CDI on participants. Amongst these quotes, the majority pertained to new course design skills or principles learned and practiced during the CDI. In particular, participants described learning about the central course design principles related to defining situational factors, writing learning objectives, aligning summative and formative assessments with content delivery, the value of instructional feedback, and incorporating educational technologies. Respondents also indicated that they gained skills and confidence in articulating, justifying, and advocating for student-centered learning practices, which they used in communicating with their supervisors, curriculum committees, or learners. Participants also described that engaging in the CDI helped them see their courses from their learners’ perspectives and to make course design decisions based on that knowledge. These responses demonstrate ways the CDI was perceived as contributing to professional development and helped foster a sense of personal accomplishment and fulfillment.

Faculty responses also indicated ways in which the CDI elicited effects that were translated to other courses and stakeholders (Table 3). For example, many of the responses on this theme described how CDI participants translated the course design skills they learned during the program to other courses they teach or how they taught their colleagues to use the course design process they learned. Additionally, participants reflected on how the CDI provided an opportunity for them to receive and give peer feedback and share practices used in their courses and disciplines with colleagues across diverse fields. Not only were the effects of the CDI felt by faculty, but respondents also shared how the knowledge they gained in the CDI contributed to improved student experiences. These outcomes ranged from increased engagement and knowledge retention to a sense of community and promotion of self-directed learning. Another collection of responses in this category pertained to CDI graduates recommending this course to and for other educators, with participants often commenting that the CDI should be required for all educators and curriculum committee members.

These positive recommendations of the CDI by participants were often accompanied by a discussion of how the CDI fit into previous faculty development opportunities they had encountered. For example, participants indicated that the CDI was valuable to those who had undergone a range of training - from prior workshops or seminars to post-professional certificate programs and advanced degrees in education (MEd or EdD). However, respondents also described opportunities for continuous improvement of the CDI. Common suggestions centered on the timing and schedule of the course as well as the need to prepare applicants with expectations for the program’s rigor. The latter of which was commonly cited in conjunction with a description of the other demands on the faculty’s time and attention. Balancing job responsibilities was also discussed by faculty in relation to whether or not they were able to translate their blueprint into action following the CDI. While many respondents indicated that they implemented their course design plan partially or in full, other responses suggested that some faculty encountered barriers (Table 3). The most commonly cited barrier was human capital; in particular, faculty felt they were unable to implement their course design ideas due to time constraints, staffing shortages, and access to technical support. Another barrier was related to budgetary constraints. However, several faculty also indicated that their plans were met with resistance from their department (supervisors, curriculum committees, course directors, etc.). This may have been due to general hesitance around change (Table 3) or some push-back around ideas that had been attempted previously (at least in part) and may not have been found to have the desired impact during the first attempt.

## Discussion

Faculty development opportunities are a commonly requested service across disciplines and can be particularly helpful for new academics and instructors transitioning from the clinic to the classroom [3, 17, 19, 32, 40, 41]. In response, the CDI model described in the present study drew upon the prior literature related to faculty development programs along with the theories of spaced repetition [5, 42–44] and situated learning [45–47] to teach medical educators the process of backwards design through a 7-week intervention where each participant applied their learning directly to their course planning. Here the program’s impact on the knowledge, skills, and attitudes of the participating faculty members was explored.

### Knowledge

The participants in this study generally entered the CDI with extensive disciplinary training but a wide range of teaching experience and formal lessons in pedagogy. Several participants indicated that they had engaged in prior faculty development or educational training (including but not limited to workshops, seminars, post-professional certificates in clinical education, and graduate-level programs in education). Nevertheless, the data from the pre−/post-tests and focus groups suggest that participants learned or deepened their understanding of topics including instructional alignment, learning goals and objectives, instructional strategies, assessment planning, feedback strategies, communication of expectations, and adult learning theories by participating in this course. These data corroborate results from other studies which have demonstrated that faculty development programs can effectively increase educators’ knowledge [48–51].

### Skills

Developing a foundation in pedagogy is a critical first step in empowering educators to design and implement student-centered instruction. Participants iteratively developed their course design plan throughout the CDI by working through a provided template (Course Design Blueprint) and incorporating feedback from peers and instructors. The final blueprint scores indicate that the CDI graduates could apply a backwards design process to their own courses. The range in scores, however, also suggests that a subset of participants experienced some barriers when developing their blueprint. A common challenge was time constraints - both internal and external to the CDI. For example, one faculty member shared that the 7-week timeframe for the course felt too short to master the material. In contrast, several others described how they experienced difficulties finding time to complete the course assignments due to other demands on their time (clinical hours, teaching load, department meetings, etc.). This sentiment is consistent with prior findings which have described time as a major barrier that prohibits faculty from engaging in professional development opportunities [32, 40]. Another challenge is that each participant had a particular context for their course design plan. For example, some focused on (re) designing seminar-style modules while others were planning 20-week courses. There was also a mix of participants who were creating courses from scratch and those who were conducting a redesign of an existing class. Additionally, certain participants were required to work within the constraints set by their departments, such as required learning objectives to meet accreditation standards; others, however, were permitted the academic freedom to explore their own pedagogical choices.

Even when challenges were encountered, participants shared how they have continued to utilize the skills they developed in the CDI. Several educators indicated that they have since used the blueprint to design additional courses they teach. Others discussed how the blueprint provided them with a straightforward process for course design that has been particularly helpful when developing instruction with a co-instructor or other stakeholders. Faculty participants also discussed how they gained perspectives by participating in this course that has allow them to improve the usability of their instruction and develop confidence in justifying and advocating for student-centered learning practices. These findings indicate how the faculty have begun translating their skills beyond this training and into their instructional practice. This transfer of developed skills is critical for empowering faculty to adopt best practices. Previous studies have demonstrated that participation in faculty development opportunities can encourage educators to reflect on their teaching practices and philosophy, develop and implement more student-centered courses, and increase the alignment between learning objectives and classroom activities or assessments [13, 22, 29, 50]. In several cases, however, the educators discussed how barriers such as human resources, departmental resistance, and budgetary constraints prevented them from immediately employing all the skills they developed in the CDI. These findings indicate how faculty development opportunities are an important component of empowering individuals to shift the educational culture at institutions. However, other factors such as buy-in from key stakeholders, protected time for course design, and access to funding for new instructional techniques or technologies are also critical. Nevertheless, when faculty were able to implement student-centered practices, they described how they noticed improvements to the student learning experience and outcomes. While the impact on students was not measured directly in the present study, descriptions from the faculty indicated that their everyday observations included increased engagement and retention of knowledge, improved ability for self-directed learning, and development of test-taking skills.

### Attitudes

Possessing knowledge and skills, however, does not necessarily correlate with motivation to apply them. Instead, self-efficacy is often used to describe the belief in one’s ability to conduct behaviors in order to achieve desired performance [52, 53]. This is also related to motivation, persistence, performance, and professional identity [53–56]. While self-efficacy is a complicated and multifaceted concept, previous literature suggests that educators with less teaching experience may also have lower self-efficacy [57, 58], which has implications for faculty development programs. Several factors including vicarious experiences, mastery experiences, feedback (also described as verbal persuasion), and emotional arousal have been identified as critical for designing interventions that can increase self-efficacy amongst participants [53, 55]. Instructors of the CDI provided vicarious experiences by modeling learner-centered instructional practices throughout the course and discussing their own teaching and learning experiences. Participants also had opportunities to engage in mastery experiences as they iteratively developed their course design blueprints and submitted a polished final version. Feedback (participant-participant and instructor-participant) and encouragement were provided frequently throughout the course via verbal dialogue, in-class activities, and written feedback on homework assignments and discussion boards. Faculty development sessions can be vulnerable spaces for participants as it can be uncomfortable to ask questions in front of one’s peers or to acknowledge what is unknown related to one’s current occupation. Therefore, the instructors endeavored to make the course engaging and dynamic while promoting a “brave” learning environment where participants were encouraged to draw from their previous experiences, discuss successes and challenges with peers, and to try new methods. Together these elements of the course may have contributed to the development of the observed self-efficacy measures and a sense of personal growth amongst the CDI participants that have been noted by faculty development programs offered through other institutions [13, 29].

Another critical outcome measure is satisfaction with the program, particularly given that participation in this CDI was voluntary. The data from both the survey and the focus groups suggested that participants were satisfied with the experience and appreciated that the course was relevant to them as educators in the health sciences and provided access to engaging instructional activities as well as resources and support in ways that scaffolded the learning appropriately. Participants noted that they would not only recommend the program to a colleague but also believe it should be a required course for all educators at the institution. Several participants remarked on how they appreciated opportunities to collaborate and learn amongst colleagues from other disciplines. Another common theme of the discussion was how this experience compared to other faculty development opportunities. The feedback suggested that the CDI was perceived as a critical learning opportunity for all participants regardless of their prior training. This finding was somewhat surprising, given that the course was designed to be an introductory course and approximately half of the participants had previously completed graduate-level coursework (including masters or doctorates of education) or post-professional certifications in education. Comments also suggested that faculty participants often felt uncomfortable providing feedback to their peers through the discussion board or peer reviews and found these activities to be some of the least effective in the course. This response was partly due to faculty feeling like they were still learning the topics themselves and therefore not feeling confident in giving feedback yet to others. These data suggest that faculty desire continuing education in pedagogy much like many healthcare practitioners are required to complete annual training in their respective disciplines.

Another common throughline in the feedback on the CDI was related to timing and balancing efforts in the class with other tasks and job responsibilities. Interestingly, while several comments described feeling like it was difficult to budget time to attend class virtually, others suggested they would like the CDI to be longer or would prefer to meet in person (which can increase engagement but also increases the time required to travel to and from class). Additionally, several respondents discussed feeling overwhelmed with completing the course planning while also being grateful for the accountability this class provided. This feedback might allude to a more profound challenge facing academics [59, 60], particularly those in the healthcare fields [61, 62], who may balance clinical duties along with other core job responsibilities including teaching, service, and research. While faculty development opportunities alone may not be a sufficient anecdote to the problem [61], it can be helpful to bring instructors together to learn from one another and to provide continuous training and support on tasks they might otherwise be tackling alone.

### Limitations and Future Directions

The present study contained several limitations and also identified future directions for continuing research. One of the major limitations is the small sample size used in this study and the demographics of this group which did not represent the diversity within the health science disciplines. Therefore, additional research is needed to explore the impacts of this CDI model in a larger, more diverse cohort and to identify whether the model is transferable to other healthcare settings. Additionally, the study was conducted at a single timepoint and from the perspective of only the CDI participants. While this offers an opportunity for the educators to reflect on their experiences in the course and how they have employed their knowledge, skills, and attitudes, it is also a limitation because there was distance between the learning intervention and several of the measured outcomes. Future research could incorporate additional instruments into the pre−/post-tests (such as the TAI) to more specifically quantify the changes promoted by the CDI and a longitudinal component might serve to elucidate the relative impact of each learning activity on the application of new course design knowledge, skills, and attitudes (including confidence, self-efficacy, and teaching philosophies). Additionally, there is an opportunity to study the impact this training had on the career outcomes and trajectories of participants. Incorporating viewpoints from various stakeholders, such as students and department chairs, could also be helpful in robustly measuring how the CDI positively impacts student skill development and learning experiences. Lastly, the study included only graduates of the program and additional insight might be gleaned from faculty members who began the program but did not complete the CDI or who chose not to enroll. Research in this area might be of particular interest, given that modest participation rates in faculty development programs are notable across higher education (and vary by demographic variables) though the reasons for this are largely unknown [32, 63]. Additional studies may also help to identify potential barriers to participation and explore the factors that contribute to motivation to engage (or not engage) in faculty development, despite evidence that supports the need for continual training that would help improve educators’ teaching knowledge, skills, and performances.

### Citation Diversity Statement

The scholarship of individuals with one or more minoritized identities are often under-cited relative to the number of manuscripts published in a given discipline [64, 65]. We believe that it is important to recognize that citation bias exists and has harmful impacts. As such, we sought to include references in this paper that reflect the diversity of scholars in this field (including, but not limited to, gender diversity, ethnicity, training, and background).

## Conclusion

The results of this study indicate that the CDI was influential in developing the faculty’s knowledge of the course design process, promoted the application of course design and pedagogy skills amongst CDI graduates, and positively impacted their self-reported attitudes about their teaching abilities. Feedback from participants demonstrates that they recognized the value of this program in their development and would recommend it to other colleagues as well. The findings suggest that providing faculty with structured, dedicated time for professional development opportunities empowered participants to learn and apply student-centered, evidence-based learning practices in their instruction in ways that can benefit the students, other faculty, and the university as a whole. Together, this study provides evidence of the efficacy for this CDI model, which can be transferable to other institutions, particularly those centered around the health sciences.

## Supplementary Information

**Additional file 1.** Additional file

**Additional file 2.** Additional file

**Additional file 3.** Additional file

## Data Availability

The data used and analyzed as part of this study are available upon reasonable request to the corresponding author.

## Abbreviations

CDI: Course Design Institute
TAI: Teaching Appraisal Inventory
