# Supplementary material for: A mixed-methods study of the effectiveness and perceptions of a course design institute for health science educators
Source: BMC Med Educ. 2022 Dec 16;22:873. doi: 10.1186/s12909-022-03910-w (PMC9756627; doi:10.1186/s12909-022-03910-w)
Supplement: Supplementary file 1 — Additional file 1. Additional file [file 12909_2022_3910_MOESM1_ESM.pdf]

ATSU | Teaching & Learning Center

Course Design Blueprint

Project Overview	
Your name:	*[Preferred Name]*
Your email:	*[YourName@college.edu]*
Course title:	*[If known, include the course and/or section number]*
Course format:	*[Online, In-person, Hybrid]*
Course stakeholder(s):	*[Co-Instructors, Subject Matter Experts, Administrators]*

Instructor's Document Approval

After an individual reviews all the details in this document, they will clearly state the revisions necessary to create a final document.

Date:

- ☐ Approved: No revisions
- ☐ Approved: With revisions noted
- ☐ Not Approved: Additional review needed

Document History

Date	Action	Name
	Initial Draft	*[Document owner]*
	Peer Review 1	*[Peer reviewer 1]*
	Peer Review 2	*[Peer reviewer 2]*
	Peer Review 3	*[Peer reviewer 3]*
	Final Review	*[Course instructor]*

Section A: Course Foundation

The first segment of this blueprint focuses on gaining a broad view of the course that will serve as the foundation for all elements to be built on.

Week 1 Part A: Course Overview

The following section provides the foundational information about the segment of instruction.

Course Synopsis	
Course description:	*[Provide a general overview of the segment of instruction. Include details if part of a larger course.]*
Course duration:	*[Describe approximately how long it will take for learners to complete the segment of instruction (course). Include details if part of a larger course.]*

Week 1 Part B: Learning Context

The following section provides key information about how the instructional content will lend itself to the learner.

Situational Factors	
Learners:	*[Who will the instruction be designed for (Students, Patients, Clinicians, Employees, etc.)? Are there any demographic factors that will influence the delivery of the instruction?]*
Location and population size:	*[Size of target audience; Where will participants be when participating in the instruction?]*
Learner aptitude regarding instructional content:	*[What is the learner's experience and attitude towards instruction in general (this particular course topic or instruction in general)?]*
Learning environment:	*[What resources will learners need to participate in this class? What will they need to have access to before, during, and/or after the instruction?]*
Digital literacy skills:	*[What technological skills will learners have prior to instruction?]*
Course pre- or co-requisites:	*[What other instruction accompanies this course that learners will need to know to be successful in this course?]*
Are there any professional or compliance standards for this instruction?	*[What standards will the learners be held accountable for with respect to the instruction (i.e., APTA, ADA, etc.)?]*
Are there any terms or acronyms that the learners may not be familiar with that might cause confusion?	*[What terminology and jargon will the learners (or other stakeholders) need to be familiar with for this course?]*

Week 2: Learning Outcomes

The following section serves as the backbone about the segment of instruction.

Targeted Outcomes	
Learning goal:	*[What is the specific goal for the segment of instruction. What should learners be able to do or know by the end of the instruction?]*
Learning objectives:	*[What are the specific learning objectives aligned to the learning goal for the segment of instruction? Include a brief description of the framework used to write them (i.e., 3-component objectives, SMART objectives, CALM, etc.). What learning domain(s) will the instruction address (i.e., Bloom's Taxonomy (factual, conceptual, procedural, metacognitive), Fink's Taxonomy (knowledge, application, integration, human, care, learning to learn)), etc.?]*

Week 3: Instructional Strategy

The following section describes the intended teaching and learning approach for the course. Being explicit about the instructional strategy will help identify the tools needed to support the learners during the educational process. This will also help ensure an evidence-based approach to learning will be used.

Pedagogical Approach	
Instructional strategy:	*[Justification of application of learning theory, tools, and techniques; e.g., self-directed, problem-based learning, team-based learning, flipped classroom, etc.]*
Scholarly references:	*[List of references used to justify the selected strategies or other resources used to develop this plan.]*

Section B: Instructional Activities

This section focuses on developing the instructional activities and flow of your course, along with developing samples of your assessment instruments.

Week 4: Content Delivery

A content outline is included below along with prompts about the related learning objectives, topics, and resources you will use for this section of instruction. The sequence should present the content in a way that's logical for your learners.

**Content Outline and Sequence**	
Course outline:	*[List the sequence of instructional content with corresponding learning objectives for the segment of instruction you will focus on. For a standard format, you can use the organizational structure with three color-coded levels:* ***Unit****,* ***Topic****, and* ***Assessment****; or you can use Fink's Strategy (2003) of In-class or Outside-of-class Activities format]*
Content materials and delivery:	*[Instructional content can consist of anything from textbooks to images (e.g., references, links to multimedia, etc.). In other words, anything you might use to construct this unit of instruction should be listed here for reference. This information can also serve as source content (study materials) for your learners, as well.*<br><br>*List the tools that will be used to create and disseminate the instructional content (i.e., LMS, video production software, 3rd party software, websites, etc.)? If appropriate, include the specific features of the tools you will use.]*

Week 5 Part A: Assessment Plan

The following section describes the assessment methods for your course. Being explicit about the assessment techniques will help identify the instruments you will use to measure whether the learners have achieved the learning objectives.

Assessment Techniques	
Assessment instruments:	*[Description of the summative and formative assessment methods and/or instruments that will be leveraged for this segment of instruction.]*
Assessment delivery:	*[What tools will be used to create and disseminate the assessment (i.e., LMS, quiz makers, simulations, websites, etc.)? How will students access the assessments?]*

Week 5 Part B: Assessment Instruments

Here, the alignment between the assessment and learning objectives will be made explicit. *Therefore, you should expect to have at least one assessment instrument or item for every learning objective.*

**Assessment Samples**	
**Assessment 1**	
Learning objective	Overview of Assessment
[Learning objective(s) aligned to this assessment.]	*[Summary of assessment technique(s) that will be used, including how will you know if the learning objective has been achieved and to what extent.]*
[Sample of assessment item (i.e., test questions, grading rubric, observation checklist, role-play scenario, etc.]	
**Assessment 2**	
Learning objective	Overview of Assessment
[Learning objective(s) aligned to this assessment.]	*[Summary of assessment technique(s) that will be used, including how will you know if the learning objective has been achieved and to what extent.]*
[Sample of assessment item (i.e., test questions, grading rubric, observation checklist, role-play scenario, etc.]	
**Assessment 3**	
Learning objective	Overview of Assessment
[Learning objective(s) aligned to this assessment.]	*[Summary of assessment technique(s) that will be used, including how will you know if the learning objective has been achieved and to what extent.]*
[Sample of assessment item (i.e., test questions, grading rubric, observation checklist, role-play scenario, etc.]	

Week 6: Instructional Alignment

In agreement with the learning science, the components of effective instruction are:

Learning Objectives + Content + Practice + Assessment + Feedback = Targeted Learning Outcomes

As such, for this component of the blueprint you will summarize all of the important instructional decisions you have made in the previous sections to highlight the alignment between all of the critical elements of your course. Using the customizable table, focus on the following components below:

1. **Learning Goal:** What is the specific goal for the segment of instruction. What should learners be able to do or know by the end of the instruction?
2. **Learning Objective:** Outcome statement that captures specifically what knowledge, skills, and attitudes learners should be able to exhibit following your course.
3. **Learning Domain:** What learning domain(s) will the instruction address according to Bloom's or Fink's Taxonomies?
4. **Instructional Strategy:** What techniques will you use to help students become independent, intentional learners?
5. **Content Delivery:** What methods will you use to disseminate the content for information related to each learning objective?
6. **Formative Assessment:** What methods will you use to assess student comprehension, learning needs, and academic progress during your course?
7. **Summative Assessment:** How will you evaluate student performance and whether or not the corresponding learning objective has been achieved?
8. **Feedback Approach:** What practical approach(es) will you use to solicit and provide feedback or guide students' reflections during your course?

**Instructional Map**						
**Learning Goal:** *[Re-statement of learning goal from Section A.]*						
**Learning Objective**	**Learning Domain**	**Instructional Strategy**	**Content Delivery**	**Formative Assessment**	**Summative Assessment**	**Feedback Approach**
[LO 1]						
[LO 2]						
[LO 3]						
[LO 4]						
[LO 5]…						

Section C: Course Improvement

The final segment of the template focuses on methods to implement your course design blueprint, as well as ways you will improve the course.

Week 7 Part A: Course Analysis

This section describes a proposed approach for how assessment results will be synthesized into an overall rating from the student and instructor perspectives.

Analysis Strategy	
Course evaluation criteria:	*[How will the assessment results be used to evaluate the student's performance? How will the results be used to quantify how well the course is working?]*

Week 7 Part B: Course Implementation*

To help you implement your course plan using this blueprint, here is an example of a Course Design Checklist of steps to follow. Additionally, below, you will find a checklist that will help you build out your course in Canvas.

Canvas Checklist				
**Communication**<br>❑ Announcements<br>❑ Notification Settings<br>❑ Chat<br>❑ Calendar	**Content Delivery**<br>❑ Video Lectures:<br>❑ Zoom<br>❑ Echo360<br>❑ Course Files	**Assessment**<br>❑ Discussions<br>❑ Quizzes<br>❑ Assignments<br>❑ Groups<br>❑ Rubrics	**Feedback**<br>❑ Gradebook<br>❑ SpeedGrader	**Other**<br>❑ Course Settings

* This Checklist was developed during the COVID-19 pandemic and therefore focused on online instruction

Week 7 Part C: Course Reflection

This section provides an opportunity for you to reflect on the feedback you have received over the iterative process of developing your instructional plan using this blueprint. Additionally, this is a space to consider what changes you might consider for the future.

Implementation of Feedback	
Summary of feedback:	*[Overall description of instructor and peer feedback you've received.]*
Summary of re-design decisions:	*[Explanation of the changes that might be implemented based on the feedback you have received.]*

Course Design Blueprint Rubric

This rubric will be used for evaluation of your Course Design Blueprint.

Grading Rubric for this Assignment<br>(Grading Scale: 2 = Good, 1 = Needs Improvement, 0 = Not present/off topic)	
**Grading Criteria**	**Score**
**Clarity: (2 Points)**<br>● Is the plan easily understood?<br>● Does it include enough detail? Is it well-organized?	
**Accurate: (2 Points)**<br>● Does the plan make correct use of instructional strategies?	
**Congruent: (2 Points)**<br>● Is the plan internally and externally consistent?	
**Thoughtful: (2 Points)**<br>● Does the plan show conscious decisions that are based on careful consideration of learning context?	
**Careful: (2 Points)**<br>● Is the plan devoid of errors in spelling, punctuation, word use, syntax, etc.?	
**Compliant: (2 Points)**<br>● Does the plan include work that is original – substantially their own?<br>● Are proper references given and appropriate permissions granted?	
**Selection: (2 Points)**<br>● Is the plan appropriate and well justified for the intended audience and purpose?	
**Instrumentation: (2 Points)**<br>● Is the assessment plan complete, authentic, practical?	
**Alignment: (2 Points)**<br>● Is each assessment instrument/item consistent with the goal or objective(s)?	
**Complexity: (2 Points)**<br>● The plan goes beyond just the superficial and instead focuses on measuring knowledge and skills (clinical practice, employable skills, mastery, etc.).	
**Total points (20):**	
