# Supplementary material for: A mixed-methods study of the effectiveness and perceptions of a course design institute for health science educators
Source: BMC Med Educ. 2022 Dec 16;22:873. doi: 10.1186/s12909-022-03910-w (PMC9756627; doi:10.1186/s12909-022-03910-w)
Supplement: Supplementary file 2 — Additional file 2. Additional file [file 12909_2022_3910_MOESM2_ESM.pdf]

Instruments Utilized in Post-Course Research Survey and Focus Groups

Self-efficacy Instrument Modified from the Teaching Appraisal Inventory from Palmer et al. (2016)

Instructions: The following questions contain a number of statements with which some people agree and others disagree. Please rate how much you personally agree or disagree with these statements to indicate how much they reflect how you feel or think personally. Use the following scale:

Response options:
1 – Strongly disagree
2 – Disagree
3 – Somewhat disagree
4 – Neither agree or disagree
5 – Somewhat agree
6 – Agree
7 – Strongly agree

Goals and objectives

1. I think I can clearly state the objectives of the class to my students
2. I think I can show students that I care about their achievement
3. I think I can stimulate students' interest in the subject area
4. I think I can increase students' interest in the course I am teaching

Assessment

5. I think I can integrate different techniques to assess students' learning
6. I think I can provide clear feedback to my students on their progress
7. I think I can design assessments to accurately measure students' performance
8. I think I can implement fair evaluation to assess student learning

Classroom environment

9. I think I can establish a good rapport with my students
10. I think I can provide help to students outside of the class period
11. I think I can create a learning environment that fosters motivation for even the unmotivated students
12. I think I can design a course where students can find answers to their questions easily
13. I think I can be helpful when students have problems with the course material

Learning activities

14. I think I can provide students with authentic examples to enhance their learning
15. I think I can integrate technology in my teaching that enhances my students' learning
16. I think I can organize my teaching or coaching to facilitate student learning
17. I think I can discuss current research related to my teaching practices
18. I think I can present the material in a way that is easy for students to understand
19. I think I can easily apply new teaching methods to better meet my students' needs
20. I think I can easily use different instructional strategies to teach
21. I think I can use alternative techniques to identify when students are confused
22. I think I can organize class activities in a way that reduces frustration
23. I think I can design my course activities that align with the learning objectives

Class facilitation

24. I think I can facilitate class discussions effectively in-person or online
25. I think I can keep the class on task during class sessions
26. I think I can effectively answer students' questions related to the class content
27. I think I can encourage students to ask questions related to the class material

28. I think I can maintain my enthusiasm in teaching even if the students do not seem to be interested in the material
29. I think I can encourage my students to express their ideas in class
30. I think I can use alternative examples to further explain the subject when students are confused
31. I think I can hold students' attention during class
32. I think I can conduct my class in an energetic way
33. I think I can manage disruptions during class effectively
34. I think I can manage technological disruptions well when I teach

Effective assignments

35. I think I can provide effective class assignments in which students collaborate with each other
36. I think I can provide students with assignments that facilitate their understanding of the material
37. I think I can assign students reading/assignments that are valuable to their learning
38. I think I can lead students to apply their learning to novel situations

Overall teaching

39. I think I can promote students' learning
40. I think I can stimulate students' thinking
41. I think I can enhance students' learning
42. I think I can help students develop their critical thinking
43. I think I can explain the course material very well
44. I think I can handle conflicts with students efficiently
45. I think I can use technology to enhance the effectiveness of my teaching well
46. I think I can teach well overall

<u>Planned Questions for the Online, Synchronous Focus Groups</u>

1. Having re-read your blueprint, what resonated with you and how do you feel about the course design plan you developed?
    a. What elements of it were the most challenging to work on?
    b. What elements were the easiest for you?
2. Have you taught the course you designed during the CDI?
3. Which elements of your blueprint have you implemented (or do you plan to implement) when teaching the course you outlined during the CDI?
    a. Which elements of your blueprint are you having trouble implementing or chosen not to implement?
4. What challenges or barriers did you face in implementing your course blueprint?
5. Compared to what you wrote in your blueprint, what elements did you change about the design when you taught the course and why?
6. What have you noticed about your students learning in your courses since participating in the CDI?
7. Are you considering making additional changes to your courses in the future? If so, what kinds of changes are you considering, and has the CDI influenced you in how you think about your student-centered teaching strategies?
8. Did your participation in the CDI impact how you thought about other courses that you are teaching? If so, in what ways?
9. What did you learn in the course design institute that you continue to use/think about/reflect on?
10. Is there anything else that you'd like to share about implementing what you learned in the CDI that we haven't asked about?
