# Supplementary material for: A mixed-methods study of the effectiveness and perceptions of a course design institute for health science educators
Source: BMC Med Educ. 2022 Dec 16;22:873. doi: 10.1186/s12909-022-03910-w (PMC9756627; doi:10.1186/s12909-022-03910-w)

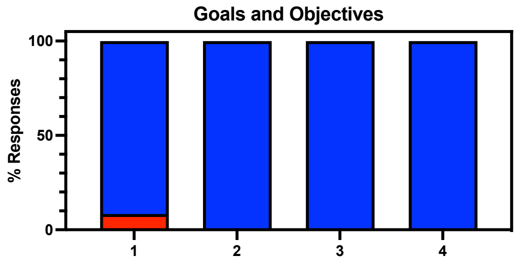

1. I think I can clearly state the objectives of the class to my students
2. I think I can show students that I care about their achievement
3. I think I can stimulate students' interest in the subject area
4. I think I can increase students' interest in the course I'm teaching

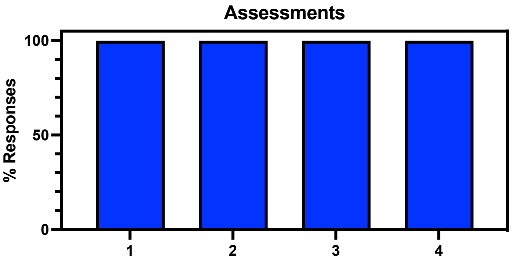

1. I think I can integrate different techniques to assess students' learning
2. I think I can provide clear feedback to my students on their progress
3. I think I can design assessments to accurately measure students' performance
4. I think I can implement fair evaluations to assess student learning

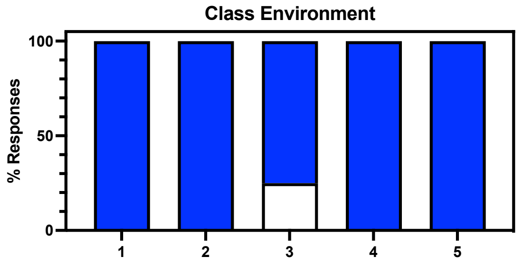

1. I think I can establish a good rapport with my students
2. I think I can provide help to students outside of the class period
3. I think I can create a learning environment that fosters motivation for even the unmotivated students
4. I think I can design a course where students can find answers to their questions easily
5. I think I can be helpful when students have problems with the course material

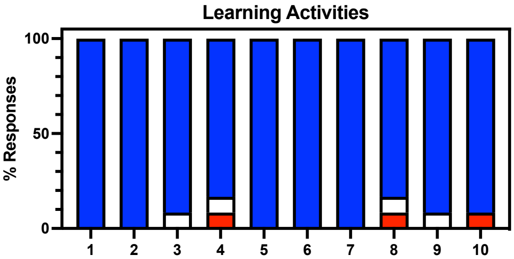

1. I think I can provide students with authentic examples to enhance their learning
2. I think I can integrate technology in my teaching that enhances my students' learning
3. I think I can organize my teaching or coaching to facilitate student learning
4. I think I can discuss current research related to my teaching practices
5. I think I can present the material in a way that is easy for students to understand
6. I think I can easily apply new teaching methods to better meet my students' needs
7. I think I can easily use different instructional strategies to teach
8. I think I can use alternative techniques to identify when students are confused
9. I think I can organize class activities in a way that reduces frustration
10. I think I can design my course activities to align with the learning objectives

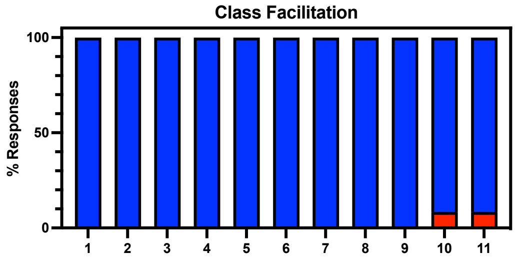

1. I think I can facilitate class discussions effectively in-person or online
2. I think I can keep the class on task during class sessions
3. I think I can effectively answer students' questions related to the class content
4. I think I can encourage students to ask questions related to the class material
5. I think I can maintain my enthusiasm in teaching even if the students do not seem to be interested in the material
6. I think I can encourage my students to express their ideas in class
7. I think I can use alternative examples to further explain the subject when students are confused
8. I think I can hold students' attention during class
9. I think I can conduct my class in an energetic way
10. I think I can manage disruptions during class effectively
11. I think I can manage technological disruptions well when I teach

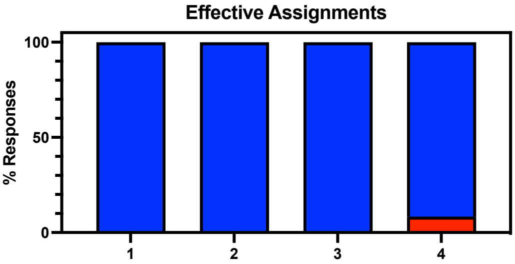

1. I think I can provide effective class assignments in which students collaborate with each other
2. I think I can provide students with assignments that facilitate their understanding of the material
3. I think I can assign students reading/assignments that are valuable to their learning
4. I think I can lead students to apply their learning to novel situations

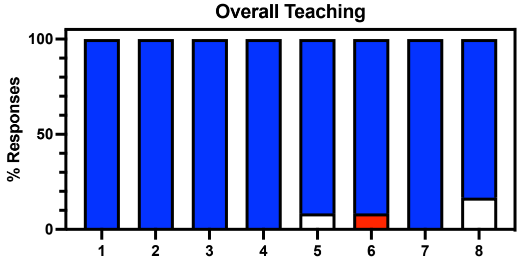

1. I think I can promote students' learning
2. I think I can stimulate students' thinking
3. I think I can enhance students' learning
4. I think I can help students develop their critical thinking
5. I think I can explain the course material very well
6. I think I can handle conflicts with students efficiently
7. I think I can use technology to enhance the effectiveness of my teaching well
8. I think I can teach well overall

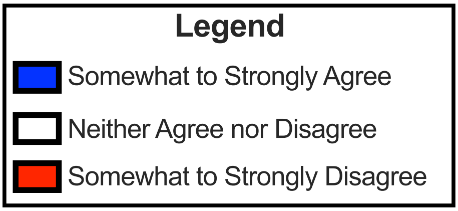

Supplement: Supplementary file 3 — Additional file 3. Additional file [file 12909_2022_3910_MOESM3_ESM.pdf]
